# In Vivo Functional Assessment of Sodium-Glucose Cotransporters (SGLTs) Using [^18^F]Me4FDG PET in Rats

**DOI:** 10.1155/2022/4635171

**Published:** 2022-06-21

**Authors:** Yohji Matsusaka, Xinyu Chen, Paula Arias-Loza, Rudolf A. Werner, Naoko Nose, Takanori Sasaki, Steven P. Rowe, Martin G. Pomper, Constantin Lapa, Takahiro Higuchi

**Affiliations:** ^1^Department of Nuclear Medicine and Comprehensive Heart Failure Center, University Hospital of Würzburg, Würzburg, Germany; ^2^Nuclear Medicine, Faculty of Medicine, University of Augsburg, Augsburg, Germany; ^3^Faculty of Medicine, Dentistry and Pharmaceutical Sciences, Okayama University, Okayama, Japan; ^4^Division of Nuclear Medicine and Molecular Imaging, The Russell H Morgan Department of Radiology and Radiological Sciences, Johns Hopkins School of Medicine, Baltimore, MD, USA

## Abstract

**Background:**

Mediating glucose absorption in the small intestine and renal clearance, sodium glucose cotransporters (SGLTs) have emerged as an attractive therapeutic target in diabetic patients. A substantial fraction of patients, however, only achieve inadequate glycemic control. Thus, we aimed to assess the potential of the SGLT-targeting PET radiotracer alpha-methyl-4-deoxy-4-[^18^F]fluoro-D-glucopyranoside ([^18^F]Me4FDG) as a noninvasive intestinal and renal biomarker of SGLT-mediated glucose transport.

**Methods:**

We investigated healthy rats using a dedicated small animal PET system. Dynamic imaging was conducted after administration of the reference radiotracer 2-deoxy-2-[^18^F]fluoro-D-glucose ([^18^F]FDG), or the SGLT-targeting agent, [^18^F]Me4FDG either directly into the digestive tract (for assessing intestinal absorption) or via the tail vein (for evaluating kidney excretion). To confirm the specificity of [^18^F]Me4FDG and responsiveness to treatment, a subset of animals was also pretreated with the SGLT inhibitor phlorizin. In this regard, an intraintestinal route of administration was used to assess tracer absorption in the digestive tract, while for renal assessment, phlorizin was injected intravenously (IV).

**Results:**

Serving as reference, intestinal administration of [^18^F]FDG led to slow absorption with retention of 89.2 ± 3.5% of administered radioactivity at 15 min. [^18^F]Me4FDG, however, was rapidly absorbed into the blood and cleared from the intestine within 15 min, leading to markedly lower tracer retention of 18.5 ± 1.2% (*P* < 0.0001). Intraintestinal phlorizin led to marked increase of [^18^F]Me4FDG uptake (15 min, 99.9 ± 4.7%; *P* < 0.0001 vs. untreated controls), supporting the notion that this PET agent can measure adequate SGLT inhibition in the digestive tract. In the kidneys, radiotracer was also sensitive to SGLT inhibition. After IV injection, [^18^F]Me4FDG reabsorption in the renal cortex was significantly suppressed by phlorizin when compared to untreated animals (%ID/g at 60 min, 0.42 ± 0.10 vs. untreated controls, 1.20 ± 0.03; *P* < 0.0001).

**Conclusion:**

As a noninvasive read-out of the concurrent SGLT expression in both the digestive tract and the renal cortex, [^18^F]Me4FDG PET may serve as a surrogate marker for treatment response to SGLT inhibition. As such, [^18^F]Me4FDG may enable improvement in glycemic control in diabetes by PET-based monitoring strategies.

## 1. Introduction

**Figure 1:**
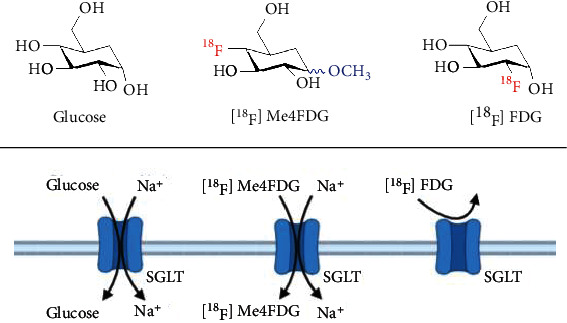
Molecular structures of [^18^F]Me4FDG, [^18^F]FDG, and glucose and their interaction with SGLT.

Recently, inhibition of sodium glucose cotransporters (SGLTs) has emerged as a therapeutic strategy for type 2 diabetes mellitus (T2DM) to increase urinary glucose excretion and/or decrease glucose absorption in the small intestine [1]. Relative to SGLT, passive glucose transporters (GLUTs) are involved in the metabolism in various other organs, including the brain, heart, and liver. Accordingly, pharmacological targeting of GLUTs for metabolic diseases is hampered due to possible off-target effects [2]. SGLTs, however, are driven by energy produced from a sodium ion gradient caused by the Na+/K+ ATPase pump. Based on this flux coupling, SGLT promotes intestinal glucose absorption via type 1 (SGLT-1), while in the kidneys, the predominantly upregulated SGLT type 2 facilitate renal glucose reabsorption in the proximal tubules. Recent major clinical trials of commercially available SGLT2-selective inhibitors have proven to substantially lower cardiovascular events in patients affected with type 2 diabetes mellitus (T2DM) [3, 4], or even had a dual beneficial effect on major cardiovascular and renal events (MARCE) in T2DM as reported by the CANVAS investigators [5]. Not surprisingly, SGLT2-selective inhibitors have been incorporated into international guidelines for the treatment of T2DM [6]. Nonetheless, a substantial portion of patients (>19%) terminated drug intake due to serious adverse events, including hypoglycemic episodes or ketoacidosis [3]. In addition, only inadequate glycemic control was achieved in high-risk patients (e.g., with preexisting renal disease) and even treatment with last-generation SGLT2 inhibitors failed to reach the recommended HbA1c target in those individuals [7]. Lifetime treatment costs are also tabulated at >$371,000, suggesting that reliable surrogate markers to identify individuals that benefit from SGLT2-selective inhibitors would be of value [8]. The positron-emitting glucose analog, *α*-methyl-4-deoxy-4-[^18^F]fluoro-D-glucopyranoside ([^18^F]Me4FDG), has already proven to provide a high affinity substrate for SGLT1/2, along with neglectable affinity for GLUTs [9, 10] (Figure 1). This is in contrast to the most commonly used glucose analog PET agent 2-deoxy-2-[^18^F]fluoro-D-glucose ([^18^F]FDG), which is characterized by a very low transport affinity for SGLTs, despite sharing structural similarities with [^18^F]Me4FDG [11]. The potential of *in vivo* [^18^F]Me4FDG PET imaging for the assessment of SGLT function had been proposed using murine and rodent models [12, 13]. First in-human studies revealed excellent image quality, with substantial low background activity [11, 14]. Here, we aimed to assess the potential of [^18^F]Me4FDG as a noninvasive intestinal and renal biomarker of SGLT-mediated glucose transport in rats.

## 2. Materials and Methods

### 2.1. Tracer Preparation

The precursor of [^18^F]Me-4FDG was purchased from ABX (ABX advanced biochemical compounds GmbH, Radeberg, Germany). The radiotracer was synthesized based on previously reported labeling protocol with modification [12]. Non-carrier-added [^18^F]F^–^ generated from cyclotron in [^18^O]H_2_O was trapped on Sep-Pak® Accell Plus QMA Plus Light cartridge (Waters GmbH, Eschborn, Germany). The [^18^F] fluoride was eluted into conical vial using a solution (250*μ*L) of 25mM K_2_CO_3_ and 50mM Kryptofix_222_ in water and acetonitrile. The eluate was concentrated and dried azeotropically with acetonitrile (500*μ*L × 2) at 120°C under nitrogen flow. A solution of the precursor (4.7mg) in dry acetonitrile (500*μ*L) was added to the residue above and reacted at 90°C for 10 min. After removing the solvent with heating and nitrogen flow, 1N HCl (500*μ*L) was added to the residue followed by further heating at 110°C for 15min. After neutralization with 1N NaOH (500*μ*L), the solution was diluted with PBS (1mL) and was purified via HPLC (Shimadzu; COSMOSIL PAQ 10 × 250mm column; mobile phase A: H_2_O, B: EtOH; 0–2min 0%B, 2–20min 30-90%B, 20–25min 90%B, 3mL/min flow rate, RT=9.2min). The collected fraction was diluted with 5% ascorbic acid in saline for further application. Thin layer chromatography (MeCN:H_2_O 85 : 15 as developing solvent, 10% sulfuric acid/ethanol as staining agent, Rf 0.83) and HPLC analysis were confirmed for radiochemical purity.

### 2.2. PET Imaging of Tracer Absorption in the Small Intestine

Animal studies were approved by the local institutional animal ethics committee and carried out in accordance with the Guide for the Care and Use of Laboratory Animals published by the U.S. National Institutes of Health (NIH publication 85-23, revised 1996) [15] and ARRIVE guidelines (https://arriveguidelines.org).

Healthy Wister rats (male; body weight 450-510 g) fasted for more than 16 hours before all experiments. The rats were anesthetized, and anesthesia was maintained with isoflurane throughout the procedures. PET imaging was performed using a dedicated small animal PET system (Inveon).

For intraintestinal tracer administration, an intestinal canulation was applied using abdominal open surgery. A tip of the microtube was inserted through the duodenum and advanced 1 to 2 cm into the small intestine. Image acquisition was started 5 min before the radiotracer administration, and list-mode data were acquired for 20 min. As a control group (*n* = 4), [^18^F]Me4FDG solution (2-4 MBq) was administered via the duodenal tube into the intestinal tracts of rats 5 min after initiation of the scan. To investigate the effect of an SGLT inhibitor on intestinal absorption of [^18^F]Me4FDG, phlorizin was administered intraintestinally and intraperitoneally (*n* = 3, each). For the intraintestinal administration, the mixture of phlorizin (4 mg/kg) and [^18^F]Me4FDG was administered into the intestinal tract via a duodenal tube. For the intraperitoneal administration, phlorizin (50 mg/kg) was administered 20 min before [^18^F]Me4FDG administration. The acquired image data were reconstructed into a dynamic sequence (20 frames: 20 × 60 s) using filtered back projection (FBP) reconstructions with decay correction. The matrix size was 128 × 128 pixels. As a comparator, [^18^F]FDG solution (3-4 MBq) was also administered (*n* = 4) in a manner similar to the controls, followed by PET acquisition.

### 2.3. PET Imaging for Renal Excretion

Fasted and anesthetized rats (male; body weight 199-213 g) were placed prone in the PET scanner, and list-mode image acquisition was started concurrently with IV administration of [^18^F]Me4FDG (41.9-46.6 MBq). To determine the specificity of [^18^F]Me4FDG kinetics and responsiveness to SGLT blockage, three groups of animals were studied, including controls (*n* = 4) and IV-pretreated animals using the SGLT inhibitor phlorizin (60 mg/kg, *n* = 4) or canagliflozin (40 mg/kg, *n* = 4; injected 20 min prior to [^18^F]Me4FDG administration). Imaging data were reconstructed into a dynamic 26-frame sequence (12 frames × 5 s, 8 frames × 30 s, 1 frames × 300 s, and 5 frames × 600 s) using FBP method with decay correction and without attenuation correction. The matrix size was 128 × 128 pixels.

Following PET imaging, the kidneys and the urine in the bladder were collected for analysis of tissue counts with an automated *γ*-counter (Wizard; PerkinElmer). Following decay correction of tissue counts, percentage of injected dose in each organ was calculated as follows:

(1) $$\begin{array}{c} \left(\%\text{ID}\right)=100\times \frac{\text{Radioactivity in organ}}{\text{Injected radioactivity}}. \end{array}$$

### 2.4. Image Analysis

**Figure 2:**
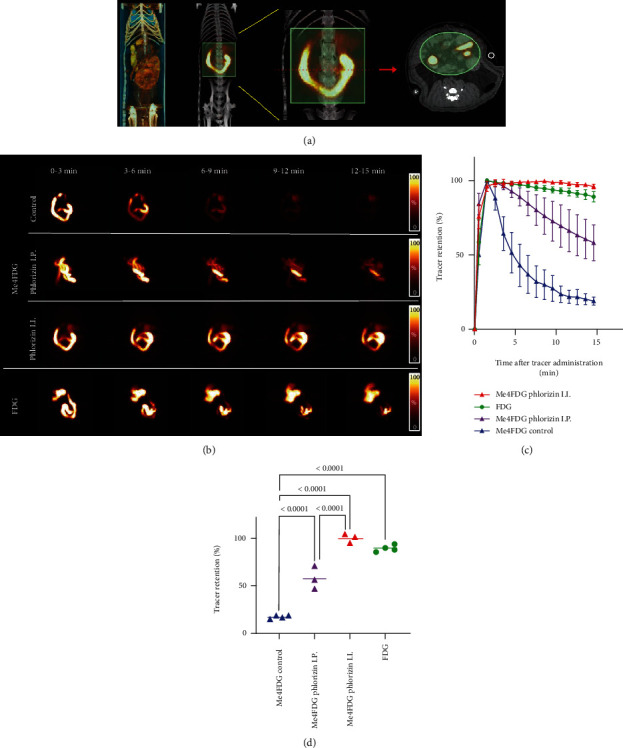
Results of intestinal [^18^F]Me4FDG PET imaging. (a) Representative volume rendered image, coronal PET/CT fusion image, and coronal and transaxial volume of interest (VOI; light green area) of a rat. (b) Coronal maximum intensity projection images of dynamic [^18^F]Me4FDG PET over 15 min after radiotracer administration in untreated controls (upper rows) and after intraperitoneal (I.P., middle rows) and intra-intestinal (I.I., lower rows) treatment using the SGLT inhibitor phlorizin. In I.I. animals treated with this drug, tracer retention remained stable, while in untreated I.I.-administered controls, a markedly reduced radiotracer accumulation was observed over time. In the last column, [^18^F]FDG serving as a comparator also demonstrated slow radiotracer clearance in untreated animals. Quantitative comparison of radiotracer retention among all groups, displayed as (c) time-activity curves and (d) tracer retention at 15 min. Control [^18^F]Me4FDG group showed rapid decrease of I.I.-injected radioactivity (blue). I.I. phlorizin-pretreated [^18^F]Me4FDG group (red) showed little decrease, similar to (untreated) I.I.-injected [^18^F]FDG animals (green, serving as reference). I.P. phlorizin-pretreated [^18^F]Me4FDG rats (purple), however, demonstrated moderate decrease during image acquisition. Bars indicate mean ± SD values.

Image analysis was conducted using the public domain program “A Medical Imaging Data Examiner” (AMIDE for Windows, version 1.0.4) [16]. In intestinal studies, fusion images of PET and CT were generated using the anatomical position of the intraluminal uptake on PET and contrast-enhanced intestinal tracts on CT. Volumes of interest (VOIs) were placed around the duodenum and whole small intestine (Figure 2(a)). Dynamic data of total radioactivity of the duodenum and small intestine were extracted. The percentage of radioactivity in each frame per maximum radioactivity after the administration was calculated, and time-activity curves were created.

In the renal studies, VOIs were placed in the cortex and pelvis of the kidneys to separate the respective signals derived from each anatomical structure. Cortex-to-pelvis count ratios were then calculated, and time-activity curves were generated.

### 2.5. Statistical Analysis

Statistical analysis was performed using GraphPad Prism 9.0 (GraphPad Software, Inc., San Diego, CA, USA). Quantitative data were expressed as mean ± SD. We conducted multiple group comparisons with analysis of variance (ANOVA) followed by Dunnett's multiple comparison test. A *P* value of less than 0.05 was considered statistically significant.

## 3. Results

### 3.1. [^18^F]Me4FDG Can Be Synthesized with High Radiochemical Purity

For the small scale manual labeling of [^18^F]Me4FDG, a solution of 0.6 equivalent of K_2_CO_3_ and 1.2 equivalent of K_222_ was used to elute the [^18^F]fluoride followed by labeling with 1.0 equivalent of precursor. As such, minimum amount of base is used to prevent the decomposition of the precursor due to extra base. The previously reported protocol was used with stacked cation/anion exchange resin column, Waters Alumina Sep-Pak, and C18 Sep-Pak cartridges after radiofluorination and deprotection of acyl groups from the intermediates [12]. However, for small radioactivity labeling protocol, the leftover on the cartridges counts was half of the final radioactivity. Therefore, the purification was modified, and the HCl was quantitatively neutralized followed by purification via HPLC with water and ethanol as eluent, so the collected fraction can be diluted and used directly for further animal studies. [^18^F]Me4FDG was obtained with comparable radiochemical yield (65% on average, *n* = 5) with a radiochemical purity of >95% with specific activity >60 GBq/*μ*mol [12].

### 3.2. [^18^F]Me4FDG Can Monitor SGLT-Mediated Inhibition of Intestinal Glucose Absorption

Representative dynamic PET images are shown in Figure 2(b). After intraintestinal injection, [^18^F]Me4FDG rapidly decreased, followed by almost complete clearance within 15 min. Both intraintestinal and intraperitoneal SGLT inhibitors, however, substantially reduced radiotracer clearance, causing signal to remain stable in the intestine up to 15 min postradiotracer injection, which was more pronounced for the intraintestinal administration route. Serving as a comparator, [^18^F]FDG also demonstrated almost no intestinal clearance. Time-activity curves of each group are shown in Figure 2(c). Radiotracer retention at 15 min in controls (18.5 ± 1.2%) was significantly lower when compared to animals with intraintestinal phlorizin treatment (99.9 ± 4.7%) (*P* < 0.0001) (Figure 2(d)). For intraperitoneally treated rats, radiotracer retention at 15 min was 58.1 ± 12.0%, which was significantly different relative to controls as well as intraintestinal inhibitor administration (*P* < 0.0001, respectively). The radioactivity of [^18^F]FDG in untreated rats confirmed slow clearance. Radiotracer retention at 15 min was 89.2 ± 3.5%, thereby demonstrating substantially elevated uptake when compared to [^18^F]Me4FDG-injected controls (*P* < 0.0001).

### 3.3. [^18^F]Me4FDG Can Monitor SGLT-Mediated Increased Renal Excretion

**Figure 3:**
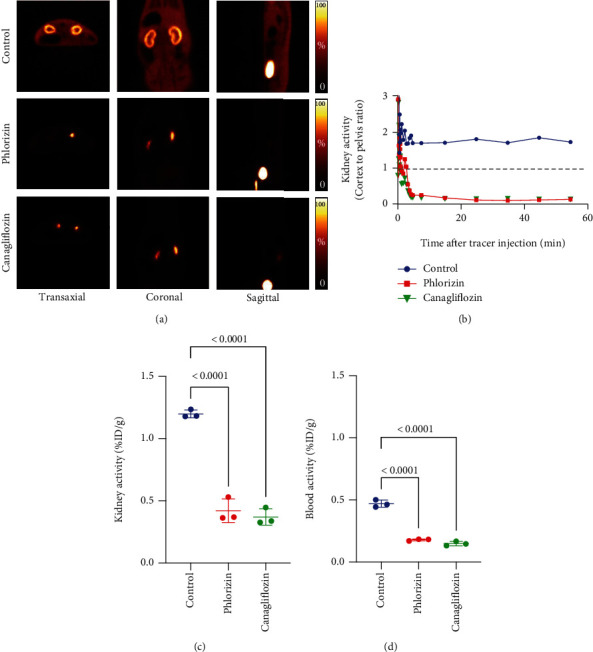
Results of renal [^18^F]Me4FDG PET imaging. (a) Multidirectional PET images of an untreated control rat versus a phlorizin- and canagliflozin-pretreated rat 10-20 min after intravenous radiotracer administration. For controls (upper rows), renal radiotracer accumulation in the cortex was substantially increased. In phlorizin-pretreated (middle rows) and canagliflozin-pretreated animals (lower rows), however, renal cortical and soft tissue activity was extremely low, while radioactivity in the renal pelvis and bladder was high. (b) Time-activity curves (cortex-to-pelvis count ratios) of all investigated rats, which revealed substantially lower renal cortical activity in pretreated animals over time. Comparison of the kidney (c) and blood radioactivity (d) at 60 min after the tracer administration among all three groups. In untreated controls, but not phlorizin- and canagliflozin-pretreated animals, the activity in the kidney and blood was substantially increased. Bars indicate mean ± SD values. %ID: percentage of injected dose.

After IV administration of [^18^F]Me4FDG, rapid radiotracer accumulation was observed in the renal cortex. This accumulation was substantially suppressed by the pretreatment of two different SGLT inhibitors, phlorizin and canagliflozin (Figures 3(a) and 3(b)). At 60 min, radiotracer activity in the control kidneys (1.20 ± 0.03%ID/g) was significantly higher than those in phlorizin- and canagliflozin-pretreated groups (0.42 ± 0.10%ID/g and 0.37 ± 0.07%ID/g; *P* <0.0001, respectively) (Figure 3(c)). In addition, the blood radioactivity in the control group (0.47 ± 0.03%ID/g) was also significantly elevated when compared to phlorizin- and canagliflozin-treated groups (0.18 ± 0.01%ID/g and 0.15 ± 0.02%ID/g; *P* < 0.0001, respectively) (Figure 3(d)).

## 4. Discussion

Using the PET agent [^18^F]Me4FDG PET and SGLT inhibitors in rats, we demonstrated functional response to SGLT blockage in the intestine and renal cortex. The dual SGLT1/2 inhibitor phlorizin almost completely inhibited the intestinal absorption of [^18^F]Me4FDG while significantly enhancing radiotracer excretion via the kidneys, which was further corroborated by the SGLT2 repressive drug canagliflozin. As such, as a noninvasive read-out of the current intestinal and renal SGLT responsiveness, [^18^F]Me4FDG PET may serve as a surrogate marker to treatment response under such inhibitory medications. In the clinic, such a PET-guided patient selection may be of relevance, as a substantial fraction of high-risk individuals frequently fail to achieve the guideline-recommended target of HbA1c levels under SGLT inhibitors [17]. In this regard, [^18^F]Me4FDG may then improve glycemic control in T2DM, as an effective dual inhibition of intestinal glucose absorption, and renal reabsorption would be characterized by high intestinal activity and low radiotracer accumulation in the renal cortex.

In T2DM patients, the recent CANVAS program reported on a reduced rate of MARCE under the SGLT inhibitor canagliflozin [5]. Despite such beneficial effects, those SGLT-inhibiting drugs are associated with high lifetime costs [8], increasing rate of adverse events including amputation [8] and suboptimal glycemic control in a substantial fraction of subjects [7]. Thus, there is an increasing demand of surrogate markers to determine patients that benefit from treatment. In this regard, a local noninvasive read-out of involved organs of SGLT-mediated glucose absorption may address this need. As such, we investigated the SGLT-targeting PET radiotracer [^18^F]Me4FDG and performed dynamic scans of both the digestive tract and the kidneys as the primary target localization of SGLT1/2 blockage [1]. [^18^F]Me4FDG was rapidly absorbed from the intestinal lumen, and the absorption was completely inhibited by simultaneously administered phlorizin (Figure 2(b)). This result is in line with the previously reported glucose absorption via SGLT1 on the intestinal epithelial surface. Using the identical experimental set-up, [^18^F]FDG absorption, however, was minimal when compared to [^18^F]Me4FDG, which also further corroborates previous findings on low SGLT1 transport affinity of [^18^F]FDG [11]. Our findings suggest that [^18^F]Me4FDG PET may provide a direct and quantitative noninvasive biomarker to monitor retention capacities of the target *in vivo*, e.g., to monitor off-target effects or to enhance therapeutic efficacy of established drugs such as (SGLT2-targeting) empagliflozin [3] or the first dual (type 1/2) inhibitor sotagliflozin [18]. Of note, the latter drug can trigger repressing effects in both organs also investigated in the present study, including delayed absorption of intestinal glucose and elevated urinary glucose excretion [18, 19]. As [^18^F]Me4FDG allowed for monitoring SGLT inhibition in the digestive (Figure 2(b)) and renal tract (Figure 3(a)), this PET agent may be particularly well suited for follow-up of D2TM patients treated with this new class of SGLT1/2 inhibition [18]. In clinical practice, after having conducted dynamic [^18^F]Me4FDG PET, that would require to draw only a few regions of interest in the renal cortex (and digestive tract), thereby providing fast quantification of SGLT responsiveness. In addition, the herein provided small animal set-up may also provide a preclinical imaging platform to assess the effects of novel SGLT inhibitors *in vivo* prior to widespread clinical adoption, which may further increase their safety margin.

In regard to the renal cortex, we demonstrated that both phlorizin and canagliflozin decreased [^18^F]Me4FDG retention in the kidney and blood, as well as enhancing urinary excretion. Those drugs inhibit SGLT2 in the proximal tubule and suppress reabsorption of glucose [5]. In control rats, [^18^F]Me4FDG accumulation was observed in the renal parenchyma where the tubules are distributed, whereas in pretreated rats, radiotracer accumulation was enhanced in the renal pelvis where it is excreted in the urine. Therefore, we calculated cortex-to-pelvis ratios to compare the renal SGLT function instead of assessing entire kidney counts, further demonstrating the high affinity of this PET agent to SGLT2.

Limitations of the present study include the use of both phlorizin and canagliflozin, while future studies may also investigate the use of other SGLT-inhibiting drugs used in clinical trials, e.g., empagliflozin [3] or sotagliflozin [18]. We should also note that certain negative controls, such as experiments for blocking of [^18^F]FDG uptake by agents such as phlorizin, were not performed, although the already-low uptake of [^18^F]FDG may mitigate the need for such experiments. Moreover, we investigated healthy animals, and additional experiments could be conducted in renal impaired and/or diabetic ZDF fa/fa rats [20], thereby closer mimicking diabetic patients with preexisting renal comorbidities that are prone to treatment failure under SGLT inhibitors [17]. By testing our experimental set-up in healthy animals, however, we provided a reliable and robust platform that could now be applied to such diabetic animal models.

## 5. Conclusions

Dynamic [^18^F]Me4FDG PET imaging identified *in vivo* SGLT function of intestinal glucose absorption and kidney excretion. As a potential noninvasive biomarker, [^18^F]Me4FDG signal may enable improvement in glycemic control in T2DM, where effective dual inhibition of intestinal glucose absorption and renal reabsorption would be characterized by high intestinal and low renal cortical radiotracer accumulation. Beyond such image-piloted therapeutic interventions, further implications include testing of novel SGLT-inhibitory drugs by using this imaging platform.

## Data Availability

The datasets generated and/or analyzed during the current study are available from the corresponding author on reasonable request.
